# Effects of baclofen on swallow motor pattern

**DOI:** 10.3389/fneur.2025.1526453

**Published:** 2025-02-25

**Authors:** Nathan W. Fielder, Michael Frazure, Ivan Poliacek, Donald C. Bolser, Kimberly E. Iceman, Teresa Pitts

**Affiliations:** ^1^School of Medicine, University of Louisville, Louisville, KY, United States; ^2^Department of Physiology, College of Medicine, University of Arizona, Tucson, AZ, United States; ^3^Department of Physiological Sciences, College of Veterinary Medicine, University of Florida, Gainesville, FL, United States; ^4^Comenius University in Bratislava, Jessenius Faculty of Medicine in Martin, Institute of Medical Biophysics, Martin, Slovakia; ^5^Department of Speech Language and Hearing Sciences and Dalton Cardiovascular Research Center, University of Missouri, Columbia, MO, United States

**Keywords:** swallow, baclofen, GABAB, laryngeal, diaphragm

## Abstract

Baclofen is a GABA_B_ receptor agonist used clinically to manage spasticity. It has also been associated with increased duration of mechanical ventilation and rates of aspiration pneumonitis. We hypothesized that baclofen would impair pharyngeal swallow, a vital airway protective reflex. Electromyography (EMG) activity was recorded in four spontaneously breathing, sodium pentobarbital-anesthetized cats. Swallow was stimulated by oral water infusion before and after administration of 3 μg/kg and 10 μg/kg (±)baclofen doses. Swallow-related thyrohyoid EMG amplitude increased after 3 μg/kg and 10 μg/kg (±)baclofen, while parasternal EMG amplitude decreased after 3 μg/kg (±)baclofen. Geniohyoid, thyroarytenoid, and thyropharyngeus EMG amplitudes increased on average, but did not reach significance. Clinically, increased thyrohyoid activation may extend duration of laryngeal abduction. Decreased parasternal activation could impair development of the negative intrathoracic pressure that aids bolus propulsion during swallow. These changes may reflect increased risk of aspiration, and more work is needed to study the effects of baclofen on airway protection.

## Introduction

Swallow is a complex and highly coordinated behavior consisting of oral, pharyngeal, and esophageal phases (1). A bolus passes through the oral cavity, reaching the oropharynx and stimulating swallow initiation. Airway closure and concomitant contraction of pharyngeal constrictor muscles transports the bolus into the esophagus, aided by inspiratory muscle activity (diaphragm and parasternal) which produces negative intrathoracic pressure during swallow (called “Schluckatmung”) (2, 3). The esophagus then directs the bolus into the stomach. The circuits that orchestrate motor control of the numerous muscles required for this behavior consist of various brainstem nuclei (2, 4). A web of brainstem premotor nuclei communicates to multiple motor nuclei in the brainstem and spinal cord and their corresponding motor units via trigeminal, hypoglossal, vagal, phrenic, and other nerves. The pharyngeal phase of swallow is noteworthy because it centers on the intersection the of airway and digestive tracts at the level of the larynx. This necessitates intricate coordination between swallow and breathing (5). Without proper coordination, dysphagia and aspiration can occur, increasing infection risk and decreasing quality of life and health outcomes (6).

Baclofen, a GABA_B_ agonist with a poorly understood mechanism of action, has a wide range of uses as an antispastic medication: clonus, flexor spasms, sequalae of spinal cord injury, cough, and alcohol withdrawal syndrome (7). It has also been used clinically as an antitussive agent because of its antispastic properties (8), and has been demonstrated to alter muscle activity during cough in cats (9). Straus et al. (10) speculate that overall, baclofen worsened breathing instability in humans by increasing variability of ventilatory periods and tidal volume. Baclofen dose is correlated with aspiration pneumonitis and both the length of mechanical ventilation and the length of hospital stay (11), even in patients who did not undergo surgery. Baclofen may also modulate the threshold for bronchoconstriction (12). There are few studies on the effects of baclofen on swallow, particularly the pharyngeal motor components, thus we recorded activity of swallow-related pharyngeal muscles to test the hypothesis that baclofen modulates swallow.

## Methods

Experiments were performed on 4 spontaneously breathing adult male cats (5.1 ± 0.6 kg). The protocol was approved by both the University of Florida and University of Louisville Institutional Animal Care and Use Committees (IACUC). The animals were initially anesthetized with sodium pentobarbital (Lundbeck, Inc., Deerfield, IL) (35 mg/kg i.v.); supplementary doses were given as needed (1–3 mg/kg i.v.). The right femoral artery and vein were cannulated to monitor blood pressure and administer intravenous fluids, respectively, and a tracheostomy was performed. Physiologic levels of end-tidal CO_2_ (4–4.5%; Datax Engstrom; Datax Ohmeda, Inc.; Madison, WI), body temperature (Homeothermic Blanket Control Unit; Harvard Apparatus; Holliston, MA), and arterial blood gas composition (i-STAT1; Abaxis; Union City, CA) were monitored and maintained (13).

Electromyograms (EMGs) were recorded using bipolar insulated fine wire electrodes (A-M Systems stainless steel Teflon coated 0.003″, bare 0.0055″, half hard), with two independent wires inserted in into each muscle for differential recordings, using the technique of Basmajian and Stecko (14). Five muscles were used to evaluate swallow motor pattern: mylohyoid, geniohyoid, thyrohyoid, thyroarytenoid, and thyropharyngeus; the parasternal muscle evaluated breathing. Both EMG placement and detection of swallow were conducted as previously described in detail (13). Swallow was induced by introducing 3ccs of water into the oropharynx via a 1-inch long, thin polyethylene catheter (outer diameter 0.5–1.0 mm) attached to a syringe.

“Spike2” was used to record and analyze EMG activity (Cambridge Electronic Design, United Kingdom). EMGs were rectified and moving averages (time constant 20 ms) obtained. Durations were measured between the swallow onset and the point where the signal returned to baseline (ms). EMG amplitude measures were normalized to the maximum amplitude that each muscle displayed during swallow and are presented as percent of maximum (i.e., maximum amplitude is 100%).

### Conditions

There were 3 conditions for all animals: the control condition with no baclofen, 3 μg/kg IA, and 10 μg/kg IA (+7 μg/kg for cumulative dosing) (±)baclofen. Doses were separated by 10 min to allow for distribution of the drug and completion of all stimulus trials.

### Statistical analysis

Means ± standard deviations were calculated and averaged for each condition across animals. Statistical differences were assessed with ANOVA (one-way repeated measures) and Tukey’s multiple comparison tests. Swallow number per trial and swallow-breathing coordination were assessed with Wilcoxon tests. An assigned coding system was used for the breathing phase in which the swallow occurred: inspiration (I; start to peak parasternal activity) as “1”; early expiration (E1; peak to end parasternal activity) as “2”; and late expiration (E2; end of parasternal activity to start of next breath parasternal activity) as “3.” For all tests, *p* < 0.05 was considered significant.

## Results

**Figure 1:**
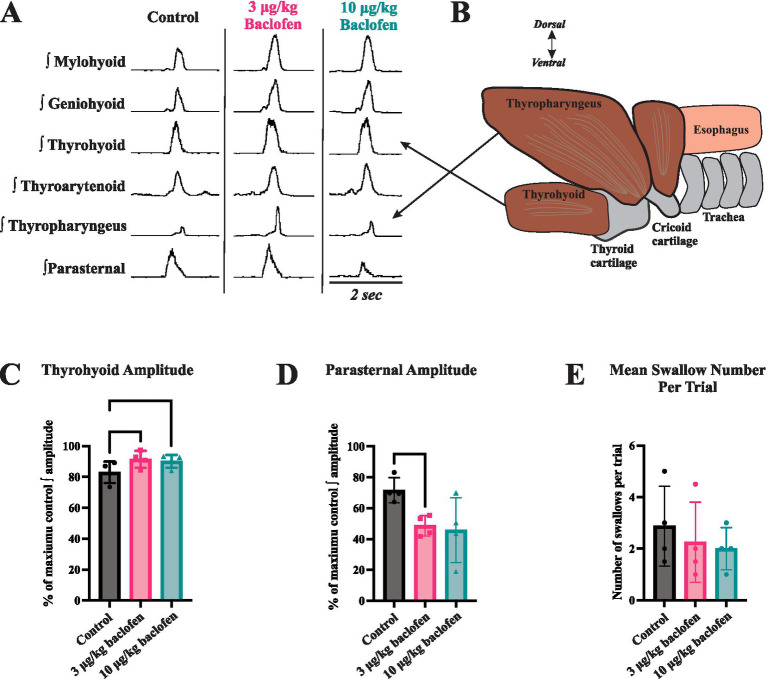
The effects of baclofen on swallow-related muscle activity. **(A)** Representative traces of muscle electromyography (EMG) recordings during swallow before (control) and after administration of 3 μg/kg and 10 μg/kg doses of (+)baclofen (signals have been rectified and integrated). **(B)** The thyrohyoid muscle attaches to the hyoid bone superiorly and thyroid cartilage inferiorly. Thyrohyoid contraction elevates the larynx and aids in closure of the epiglottis over the airway. **(C)** Thyrohyoid EMG amplitude increased following administration of 3 μg/kg and 10 μg/kg (±)baclofen. **(D)** Parasternal EMG amplitude decreased following administration of 3 μg/kg (±)baclofen. EMG amplitudes were normalized to maximum for statistical comparisons. **(E)** Swallow number per stimulus trial showed a decreasing trend with baclofen, but this was not significant.

Swallow was reliably elicited during the entire protocol in all animals. Figure 1 illustrates the effect of (±)baclofen on EMG amplitudes of swallow-related muscles. It significantly increased the maximum EMG amplitude of the thyrohyoid (*p* = 0.004) with 3 μg/kg (*p* = 0.007) and 10 μg/kg (*p* = 0.04) doses compared to control, and decreased parasternal EMG amplitude with the 3 μg/kg dose (*p* = 0.009) compared to control. Results are reported in Table 1. Geniohyoid, thyroarytenoid, and thyropharyngeus EMG amplitudes increased on average, but did not reach significance. Following baclofen administration, there were no significant changes in muscle EMG durations, swallow duration, nor swallow number per stimulation trial. A Wilcoxon test showed no difference in swallow-breathing coordination between control and 3 μg/kg (Z = −0.17, *p* = 0.9) or 10 μg/kg (*Z* = −1.16, *p* = 0.25) (±)baclofen conditions.

**Table 1:** The effects of baclofen on swallow.

	Control	Baclofen 3 μg/kg	Baclofen 10 μg/kg	*p*-value*		
	Mean ± SD	Mean ± SD	Mean ± SD	Ctrl vs. 3 μg	Ctrl vs. 10 μg	3 μg vs. 10 μg
Amplitude (% max)						
Hyoid/Laryngeal Elevators Mylohyoid	83 ± 10	85 ± 13	84 ± 9	0.96	0.99	0.90
Geniohyoid	79 ± 9	85 ± 9	87 ± 5	0.23	0.37	0.93
Thyrohyoid	83 ± 7	91 ± 5	90 ± 4	**0.007**	**0.04**	0.62
Pharyngeal Thyropharyngeus	64 ± 27	67 ± 13	79 ± 7	0.98	0.54	*0.07*
Laryngeal Adductor Thyroarytenoid	72 ± 13	77 ± 17	81 ± 17	0.40	0.55	0.82
Inspiratory Parasternal	72 ± 8	49 ± 7	46 ± 21	**0.009**	0.28	0.97
Duration (ms)						
Mylohyoid	520 ± 124	518 ± 97	512 ± 48	0.99	0.98	0.97
Geniohyoid	468 ± 78	451 ± 56	433 ± 31	0.70	0.70	0.81
Thyrohyoid	452 ± 143	428 ± 56	416 ± 62	0.86	0.75	0.77
Thyropharyngeus	479 ± 105	428 ± 35	430 ± 55	0.43	0.35	0.99
Thyroarytenoid	484 ± 148	463 ± 71	454 ± 66	0.91	0.76	0.90
Swallow duration	548 ± 127	514 ± 68	515 ± 59	0.64	0.63	0.99
Swallow number per trial	2.9 ± 1.5	2.3 ± 1.6	2.0 ± 0.8	0.13	0.13	0.13

*p-value ≤ 0.05 in bold. *p-value approaching significance in italics.

## Discussion

Our results demonstrate that swallow motor patterns are altered following baclofen administration. To our knowledge, our study is the first to evaluate effects of baclofen on the motor pattern and durations of oropharyngeal muscles during swallow. Clinical literature demonstrates increased rates of aspiration pneumonitis and extension of mechanical ventilation among patients on baclofen (10, 11). Baclofen-induced dysphagia may play a role in these cases, but the explicit mechanism is not well studied. Typically, the thyrohyoid muscle achieves an elevation of the larynx to close the epiglottis over the airway. Increased thyrohyoid activation may discoordinate the swallow pattern to allow pharyngeal contents to pass into the larynx.

We previously reported that baclofen decreased the rectus abdominis EMG amplitude during coughing, the inspiratory and active expiratory (E1) phases of cough, and cough number per stimulus. Baclofen did not affect EMG amplitudes of laryngeal muscles or parasternal muscles, nor the duration of the passive expiratory (E2) phase during cough. This suggested that laryngeal and inspiratory motor drives during cough are controlled differentially. The current results confirm this, based on the fact that thyrohyoid amplitude increased while parasternal amplitude decreased. This is relevant for spinal cord injury wherein spinal tracts would be disrupted, while cranial pathways left intact. Collectively, these results provide evidence for a control system that regulates laryngeal activity, inspiratory spinal drive, and expiratory spinal motoneurons separately. It is possible that the increase in upper airway muscle activity during swallow that we observed in this study is a secondary consequence of reduced activity of inspiratory muscles, rather than a result of direct action on GABA receptors. The influence of baclofen on cough has also been studied in spinal cord injury (15); subjects treated with baclofen had a significantly higher cough threshold (diminished cough reflex sensitivity) than control subjects.

Baclofen exerts different effects on components of breathing drive, and this can differ by species [discussed in Straus et al. (10)]. Its overall effect in many mammal species seems to be ventilatory depression. However, baclofen has been shown to have excitatory (increased diaphragm discharge) or disinhibitory (lung inflation reflex) effects in rats (16, 17). In cat, small doses of baclofen can increase phrenic nerve discharge and tidal volume (18). Depending on the dose, baclofen can have inhibitory or excitatory effect in motoneurons (19), and does not appear to exert its antispastic action postsynaptically at a clinical dose. While GABA signaling is known to be important in central control of breathing, the central respiratory effects of baclofen have not been well studied in mammals. The site(s) of action of baclofen on breathing and in the current study are unknown. However, Straus et al. (10) concluded that baclofen interfered with central respiratory patterning in their human study, in which it worsened breathing instability.

A small number of other studies have investigated the effects of baclofen on swallow, primarily the esophageal phase. One study reported the effects of baclofen and another GABA_B_ agonist on transient lower esophageal sphincter relaxations and swallow in conscious dogs (20). Both compounds inhibited transient lower esophageal sphincter relaxations and spontaneous swallow. When swallowing was stimulated with oral water injection, both compounds also inhibited primary peristalsis. The authors concluded that these agents acted by inhibiting the swallow central pattern generator, but this could also be related to a more direct effect on esophageal autonomic circuits. Hung and colleagues (21) posit that baclofen acts to block sensory inputs to the central swallow pattern generator without altering the pharyngeal swallow motor pattern. They conducted a manometry study in healthy humans who received oral baclofen or placebo and concluded that while baclofen has little to no effect on volitional swallow, it may impair upper esophageal sphincter contractility, and impair pharyngeal swallow by reducing the effective swallowing of large volumes (piecemeal swallow). However, in another study in cats, baclofen selectively blocked the reflex responses to rapid esophageal distension and reduced water-evoked pharyngeal swallow frequency (22). We did not observe any changes in swallow number or duration or swallow-breathing coordination in the current study.

The general GABA mimetic muscimol and the GABA_A_ agonist diazepam both inhibit electrically-stimulated swallow in cat (23), and GABA_A_ activity is thought to mediate both pharyngeal and esophageal deglutitory inhibition (24). In a study in anesthetized rats, swallows were evoked by pharyngeal distension, punctate mechanical stimulation, capsaicin, distilled water applied topically to the vocal folds, or by electrical stimulation of the superior laryngeal nerve (25). Infusion of either baclofen or diazepam inhibited swallows evoked by mechanical, chemical, and electrical stimulation, and these effects were reversed by the respective antagonists. However, there was no significant effect of baclofen on EMG burst durations of suprahyoid and thyrohyoid muscles; burst amplitude was not reported. Collectively, these data indicate that diazepam and baclofen both centrally inhibit components of swallow.

## Conclusion

The GABA_B_ agonist baclofen enhanced thyrohyoid activation during swallow, and decreased parasternal activation. Along with our previous study of the effects of baclofen on cough, our results support the conjecture that laryngeal activity, inspiratory spinal drive, and expiratory spinal motoneurons are controlled separately. It is possible that the divergence reveals that baclofen’s greatest effects are on spinal motoneurons. In spinal cord injury, spinal tracts are disrupted, while cranial pathways are left intact, and cervical spinal cord injury produces laryngeal dysregulation and dysphagia, but the underlying mechanisms are unknown (26). Patients with spinal cord injuries are commonly treated with baclofen to reduce spasticity; however, it decreases cough reflex sensitivity (15), and can inhibit swallow even in healthy cats (23) and rats (23, 25). Future studies may investigate GABA_B_ brainstem receptors, various animal models, or patients experiencing baclofen-induced dysphagia or dystussia. Findings of disordered activation during airway protective behaviors may play a clinical role in screening/therapy for dysphagic complications and invite future studies.

## Data Availability

The raw data supporting the conclusions of this article will be made available by the authors, without undue reservation.

## References

[1] Miller AJ. Neurophysiological basis of swallowing. Dysphagia. (1986) 1:91–100. doi: 10.1007/BF02407121

[2] Pitts T Iceman KE. Deglutition and the regulation of the swallow motor pattern. Physiology (Bethesda). (2023) 38:10–24. doi: 10.1152/physiol.00005.2021, PMID: 35998250 PMC9707372

[3] Marckwald M. The movements of respiration: And their innervation in the rabbit. With a supplement on the relation of respiration to deglutition, and on the question of the existence of respiratory Centres in the spinal cord. London, UK: Blackie & Son (1888).

[4] Bieger D. Rhombencephalic pathways and neurotransmitters controlling deglutition. Am J Med. (2001) 111:85–9. doi: 10.1016/S0002-9343(01)00824-5, PMID: 11749931

[5] Martin-Harris B Brodsky MB Price CC Michel Y Walters B. Temporal coordination of pharyngeal and laryngeal dynamics with breathing during swallowing: single liquid swallows. J Appl Physiol. (2003) 94:1735–43. doi: 10.1152/japplphysiol.00806.2002, PMID: 12506044

[6] Ekberg O Hamdy S Woisard V Wuttge-Hannig A Ortega P. Social and psychological burden of dysphagia: its impact on diagnosis and treatment. Dysphagia. (2002) 17:139–46. doi: 10.1007/s00455-001-0113-5, PMID: 11956839

[7] Kent CN Park C Lindsley CW. Classics in chemical neuroscience: baclofen. ACS Chem Neurosci. (2020) 11:1740–55. doi: 10.1021/acschemneuro.0c00254, PMID: 32436697

[8] Dicpinigaitis PV Dobkin JB. Antitussive effect of the GABA-agonist baclofen. Chest. (1997) 111:996–9. doi: 10.1378/chest.111.4.996, PMID: 9106580

[9] Castillo D Pitts T. Influence of baclofen on laryngeal and spinal motor drive during cough in the anesthetized cat. Laryngoscope. (2013) 123:3088–92. doi: 10.1002/lary.24143, PMID: 23670824 PMC4936775

[10] Straus C Teulier M Morel S Wattiez N Hajage D Giboin C. Baclofen destabilises breathing during sleep in healthy humans: a randomised, controlled, double-blind crossover trial. Br J Clin Pharmacol. (2021) 87:1814–23. doi: 10.1111/bcp.14569, PMID: 32986891

[11] Pommier P Debaty G Bartoli M Viglino D Carpentier F Danel V. Severity of deliberate acute baclofen poisoning: a nonconcurrent cohort study. Basic Clin Pharmacol Toxicol. (2014) 114:360–4. doi: 10.1111/bcpt.12161, PMID: 24138484

[12] Dicpinigaitis PV Nierman DM Miller A. Baclofen-induced bronchoconstriction. Ann Pharmacother. (1993) 27:883–4. doi: 10.1177/106002809302700713, PMID: 8364269

[13] Pitts T Rose MJ Mortensen AN Poliacek I Sapienza CM Lindsey BG. Coordination of cough and swallow: a meta-behavioral response to aspiration. Respir Physiol Neurobiol. (2013) 189:543–51. doi: 10.1016/j.resp.2013.08.009, PMID: 23998999 PMC3882902

[14] Basmajian JV Stecko G. A new bipolar electrode for electromyography. J Appl Physiol. (1962) 17:849. doi: 10.1152/jappl.1962.17.5.849

[15] Dicpinigaitis PV Grimm DR Lesser M. Baclofen-induced cough suppression in cervical spinal cord injury. Arch Phys Med Rehabil. (2000) 81:921–3. doi: 10.1053/apmr.2000.5612, PMID: 10896005

[16] Seifert E Trippenbach T. Effects of baclofen on the Hering-Breuer inspiratory-inhibitory and deflation reflexes in rats. Am J Phys Regul Integr Comp Phys. (1998) 274:R462–9. doi: 10.1152/ajpregu.1998.274.2.R462, PMID: 9486305

[17] Trippenbach T Lake N. Excitatory cardiovascular and respiratory effects of baclofen in intact rats. Can J Physiol Pharmacol. (1994) 72:1200–7. doi: 10.1139/y94-170, PMID: 7882186

[18] Pierrefiche O Foutz AS Denavit-Saubié M. Effects of GABAB receptor agonists and antagonists on the bulbar respiratory network in cat. Brain Res. (1993) 605:77–84. doi: 10.1016/0006-8993(93)91358-Y, PMID: 8385542

[19] Li Y Li X Harvey PJ Bennett DJ. Effects of baclofen on spinal reflexes and persistent inward currents in motoneurons of chronic spinal rats with spasticity. J Neurophysiol. (2004) 92:2694–703. doi: 10.1152/jn.00164.2004, PMID: 15486423

[20] Lehmann A Bremner-Danielsen M Brändén L Kärrberg L. Inhibitory effects of GABA(B) receptor agonists on swallowing in the dog. Eur J Pharmacol. (2002) 448:67–70. doi: 10.1016/S0014-2999(02)01907-6, PMID: 12126973

[21] Hung JS Liang SW Omari T Wong MW Lei WY Yi CH. Effects of the GABA(B) agonist baclofen on volitional swallowing in normal subjects. Kaohsiung J Med Sci. (2023) 39:80–6. doi: 10.1002/kjm2.12607, PMID: 36245436 PMC11895948

[22] Lang IM Medda BK Shaker R. Mechanisms of reflexes induced by esophageal distension. Am J Physiol Gastrointest Liver Physiol. (2001) 281:G1246–63. doi: 10.1152/ajpgi.2001.281.5.G1246, PMID: 11668034

[23] Hockman CH Weerasuriya A Bieger D. GABA receptor-mediated inhibition of reflex deglutition in the cat. Dysphagia. (1996) 11:209–15. doi: 10.1007/BF00366388, PMID: 8755468

[24] Wang YT Bieger D. Role of solitarial GABAergic mechanisms in control of swallowing. Am J Phys. (1991) 261:R639–46. doi: 10.1152/ajpregu.1991.261.3.R639, PMID: 1653542

[25] Tsujimura T Sakai S Suzuki T Ujihara I Tsuji K Magara J. Central inhibition of initiation of swallowing by systemic administration of diazepam and baclofen in anaesthetized rats. Am J Physiol Gastrointest Liver Physiol. (2017) 312:G498–507. doi: 10.1152/ajpgi.00299.2016, PMID: 28254772 PMC6347068

[26] Pitts T Iceman KE Huff A Musselwhite MN Frazure ML Young KC. Laryngeal and swallow dysregulation following acute cervical spinal cord injury. J Neurophysiol. (2022) 128:405–17. doi: 10.1152/jn.00469.2021, PMID: 35830612 PMC9359645

